# Role of nutritional status in predicting quality of life outcomes in cancer – a systematic review of the epidemiological literature

**DOI:** 10.1186/1475-2891-11-27

**Published:** 2012-04-24

**Authors:** Christopher G Lis, Digant Gupta, Carolyn A Lammersfeld, Maurie Markman, Pankaj G Vashi

**Affiliations:** 1 Cancer Treatment Centers of America® (CTCA), 1336 Basswood Road, Schaumburg, IL, 60173, USA

**Keywords:** Quality of life, Malnutrition, Nutritional assessment, Nutritional status, Oncology

## Abstract

Malnutrition is a significant factor in predicting cancer patients’ quality of life (QoL). We systematically reviewed the literature on the role of nutritional status in predicting QoL in cancer. We searched MEDLINE database using the terms “nutritional status” in combination with “quality of life” together with “cancer”. Human studies published in English, having nutritional status as one of the predictor variables, and QoL as one of the outcome measures were included. Of the 26 included studies, 6 investigated head and neck cancer, 8 gastrointestinal, 1 lung, 1 gynecologic and 10 heterogeneous cancers. 24 studies concluded that better nutritional status was associated with better QoL, 1 study showed that better nutritional status was associated with better QoL only in high-risk patients, while 1 study concluded that there was no association between nutritional status and QoL. Nutritional status is a strong predictor of QoL in cancer patients. We recommend that more providers implement the American Society of Parenteral and Enteral Nutrition (ASPEN) guidelines for oncology patients, which includes nutritional screening, nutritional assessment and intervention as appropriate. Correcting malnutrition may improve QoL in cancer patients, an important outcome of interest to cancer patients, their caregivers, and families.

## Introduction

Disease-related malnutrition occurs frequently in patients with cancer and is a major cause of morbidity and mortality.[1] The incidence of malnutrition in cancer patients ranges between 40 and 80% [2] while the prevalence ranges from 50% to 80%[3] depending on tumor type, tumor location, stage of disease, treatment received and the type of nutritional assessment method used.[4] Decreased dietary intake, cancer cachexia (characterized mainly by weight loss and muscle wasting), and nutrition impact symptoms may all contribute to cancer-related malnutrition.[3] Additionally, the treatment modalities involving combinations of chemotherapeutic, radiotherapeutic and surgical regimens are known to produce various acute and chronic symptoms that limit eating and, thereby, exert a profound impact on nutritional status.[1,5]

As a result, it is important to assess every cancer patient’s nutritional status using one or more methodologies that are developed for this purpose. Anthropometric measurements [such as weight change, arm muscle circumference (AMC), triceps skin-fold thickness (TSF)] and biochemical parameters (such as serum albumin)[6] are often used. Other instruments, such as Subjective Global Assessment (SGA)[7] and Patient-Generated Subjective Global Assessment (PG-SGA), which has been adapted from the SGA and designed specifically for patients with cancer[3,8] are also used. Still other tools have been recommended by the European Society for Clinical Nutrition and Metabolism, including Nutritional Risk Screening (NRS-2002), which has demonstrated high sensitivity and specificity at hospital admission; the Malnutrition Universal Screening Tool (MUST), which was devised for people who live in a community setting and relates their nutrition to their functions; and the Mini Nutritional Assessment (MNA), which was designed specifically for elderly people.[9] Tools such as phase angle determined by Bioelectric Impedance Analysis (BIA) and hand grip strength measured by dynamometry are also used as markers of nutritional status.[10] The utility of these nutritional screening tools has been evaluated by their ability to predict relevant clinical outcomes such as complications, treatment response, survival and quality of life (QoL).

Quality of life for cancer patients is a subjective multidimensional construct that represents the patient’s functional status, psychosocial well-being, health perceptions and disease/treatment-related symptoms.[7] In all, QoL reflects health status in cancer patients, which, in turn, is largely influenced by nutritional factors.[9] Cancer and treatment-induced changes in metabolism can lead to alterations in physiological and psychological functions, which, in turn, can reduce a patient’s QoL by negatively influencing nutritional status.[11]

Measuring a patient’s QoL is contingent upon clinical goals and may require a variety of different instruments. Among them are the European Organization for Research and Treatment of Cancer quality-of-life core questionnaires (EORTC QLQ-C30), the Functional Assessment of Cancer Therapy-General (FACT-G), Functional Living Index cancer questionnaire (FLIC), Spitzer Quality of Life Index (QLI), Rotterdam Symptom Check List (RSCL), the Medical Outcome Study 36-item short form (MOS SF-36), EuroQol (EQ-5D)[9], the Cancer Rehabilitation Evaluation System (CARES)[12] and the Symptom Distress Scale.[13]

Researchers have studied the correlation between nutritional status and QoL for some time and there are a number of articles in the literature that provide data on this association in cancer patients. Because these studies differ from each other with respect to patient population, study design, sample size and type of nutritional and QoL assessment used, it can be difficult to interpret and synthesize common findings. Our goal was to systematically review the available literature, summarize the vast amount of information on the topic of nutrition and QoL in cancer patients, and provide direction for future research.

## Methods

### Data sources

We attempted to plan, conduct, and report this meta-analysis in accordance with the PRISMA statement.[14,15] MEDLINE database was used to identify all articles investigating the relationship between nutritional status and QoL in cancer published between January 01, 1990 and June 30, 2011. We also searched the bibliographies of selected papers to identify relevant articles we might have missed during the primary MEDLINE search.

### Study eligibility criteria

To be included in this review, an article must have: been published in English, reported on data collected in humans with cancer, had malnutrition/nutritional status/assessment/screening as one of the predictor variables, had QoL as one of the outcome measures (primary or secondary), and had any of the following study designs (case–control, cohort, cross-sectional, prospective, retrospective, case series, longitudinal, clinical trial, meta-analysis). There were no restrictions based on age, ethnicity, type or stage of cancer.

### Search

We searched the MEDLINE database using the terms “nutritional status” or “nutritional assessment” or “nutritional screening” or “malnutrition” in combination with the following terms: “quality of life”, and “health-related quality of life” together with “cancer” or “oncology”.

### Study selection

Study selection was based on an initial screen of identified abstracts or titles and a second screen of full-text articles. Articles to be included in this review were screened for eligibility by two reviewers working independently. Disagreements between reviewers were resolved by consensus.

### Data collection process

One review author extracted the required data elements from included articles and the second author verified the extracted data. Disagreements were resolved by discussion between the two review authors. Tables 1, 2, 3, 4, 5 reflect the template for data collection.

**Table 1:** Nutritional status and quality of life in head and neck cancer

**First Author, Year, Study Place**	**Data Collection Period**	**Study Design**	**Sample Size**	**Nutritional Assessment**	**Quality of Life Assessment**	**Groups being compared**	**Key results**	**Conclusion**
Jager-Wittenaar H, 2011, The Netherlands [16]	October 2004 and February 2006	Convenience sample, cross-sectional study	115 oral or oropharyngeal cancer	Percentage weight loss was calculated as: [(normal body weight - actual body weight)/normal body weight] *100	EORTC QLQ C-30	Weight loss > =10% in 6 months or > =5% in 1 month	Median scores of malnourished patients on physical functioning (p = .007) and fatigue (p = .034) were significantly lower than those of well-nourished patients.	Malnourished patients treated for oral/oropharyngeal cancer score lower on quality of life scales related to physical fitness.
Capuano G, 2010, Italy [8]	NA	Prospective, consecutive case series	61 Head & Neck Cancer Oropharynx: n = 21; Oral cavity: n = 19 Nasopharynx: n = 13; Larynx: n = 5; Maxillary sinus: n = 2 Submandibular gland: n = 1	1. Unintended weight loss (UWL) 2. PG-SGA score	EORTC QLQ C-30	**Unintended weight loss –** **Non-malnourished:** involuntary loss of < 5% of body weight in the last 3 months (n = 36) &**Malnourished:** ≥ 5% loss of body weight in the last 3 months (n = 25)	**1. Unintended weight loss –** **Multivariate:** Malnutrition (UWL) and Hb level independently influenced physical (p = 0.002; p = 0.005), role (p = 0.004; p = 0.001), and social functions (p = 0.024; p = 0.009). **2. PG-SGA score –** Mean ± SD = 3 ± 2 & 9 ± 5 respectively for non-malnourished & malnourished patients, p < 0.001.	An early and intensive nutritional support might reduce weight loss before, during, and after treatment completion, improving outcome, QoL, and PS.
Morton RP, 2009, New Zealand [17]	Over a 24-month period, ending in 2005	Retrospectiveconsecutive case series	36 head and neck cancer	BMI drop over 12 months	UW-QOL	BMI change was taken as a continuous variable	The 12-month BMI drop was inversely correlated with current HRQOL, signifying that weight loss correlated with a poorer subsequent HRQOL score (r = −0.47, P = 0.026). It was significantly related to lower speech and swallowing function scores.	The observed relationship between a drop in BMI and the current HR-QOL may be a function of greater general impact of treatment.
van den Berg MGA, 2007, the Netherlands [18]	May 2002 to May 2004	Observationalprospective non-randomized, longitudinal study	47 Squamous Cell Carcinoma of the oral cavity, oropharynx, hypopharynx. Oral cavity: n = 23; Oropharynx: n = 18; Hypopharynx: n = 5	Unintended weight loss Malnutrition was defined as unintended weight loss of 10% or more within the previous 6 months before baseline	EORTC QLQ C-30and EORTC QLQ – H&N35	≥ 10% & < 10% weight loss at baseline	**1. At baseline:** Patients ≥10% weight loss in 6 months before baseline had lower scores for global, physical, role, and emotional functioning. Fatigue, pain, insomnia, appetite loss, swallowing, decreased sexuality, sticky saliva and coughing were worse in the ≥ 10% weight loss group. **2. At the end of treatment:** Patients who had lost ≥ 10% weight had lower role and social functioning. Scores significantly differed for global (p = 0.01), fatigue (p = 0.03), pain (p = 0.04), senses problems (0.05), sticky saliva (p = 0.01), coughing (p = 0.02) and feeling ill (p = 0.01) during treatment. **3. Six months after treatment:** Patients ≥ 10% weight loss lower on physical, role, emotional and cognitive functioning.	Patients with head and neck cancer treated with radiotherapy are specifically susceptible to malnutrition during treatment with no improvement in body weight or QoL.
Petruson KM, 2005, Sweden [19]	February 1996 to May 1997	Prospective, longitudinal study	49 primary untreated head and neck cancer Pharyngeal: n = 15; Laryngeal: n = 12; Oral: n = 12; Other: n = 10	Weight loss* * Severe weight loss (malnutrition) defined as loss of more than 10% weight during 6 months	1. EORTC QLQ-C30 2. EORTC QLQ-H&N35 3. HADS	≥ 10% weight loss (n = 20) & < 10% weight loss (n = 29)	**(A) At different time-points:** Patients who lost ≥ 10% in weight during 6 months had worse HRQL at diagnosis than did patients who lost less at all time-points. **(B) HADS:** At diagnosis, 37% of the ≥ 10% weight loss group had Possible/probable depression versus 17% of the <10% weight-loss group. This tendency remained after 3 months (38% vs 20%), at 1-year follow-up (44% vs 5%), and after 3 years (27% vs 15%).	Patients with head and neck cancer who are at risk of severe weight loss developing during treatment may be detected with the aid of HRQL questionnaires at diagnosis.
Hammerlid E, 1998, Norway, Sweden [12]	NA	Prospective, consecutive case series	48 head and neck cancer Oral cavity: n = 16 Larynx: n = 11 Sinus: n = 10 Skin: n = 4 Esophagus/ Hypopharynx: n = 4 Other: n = 3	1. Weight loss 2. Anthropo-metry: (a) AMC and (b) TSF 3. WI 4. BMI 5. S-alb	EORTC QLQ-C30 supplemented by a provisional H&N cancer module constructed in Norway	**1. Weight loss:** > 5% & ≤ 5% of the body weight **2. Anthropometry:** Based on Swedish reference values **3. WI:** < 0.80 & ≥ 0.80 **4. BMI:** ≥ 20 & < 20 **5. S-alb:** < 33 g/L & ≥ 33 g/L **Groups for analysis** (1) malnutrition (n =25) versus normal (n = 22), (2) weight loss (n = 20) versus no weight loss (n = 24), (3) negative energy balance (n = 18) versus positive energy balance (n = 15)	**1. Malnutrition versus normal nutritional status:** Malnourished patients scored worse for 12 of the 16 functions/symptoms. The greatest differences between the two groups were found for Physical Function, global QoL, and Role Function, NS. **2. Weight loss versus no weight loss:** Patients with weight loss scored worse for 11 of 16 functions. (a) Swallowing difficulties Mean score = 52 & 18 for those with weight loss and no weight loss respectively, p < 0.01. (b) Problems swallowing food Mean score = 62 & 29 for those with weight loss and no weight loss respectively, p < 0.01. **3. Negative and positive energy balance:** The groups of patients with negative energy balance scored better than the group of patients with positive energy balance for 11 of the 16 function/symptoms, NS.	This study demonstrated few significant differences, depending on nutritional status, in some of the QL scales or item scores.

**Table 2:** Nutritional status and quality of life in gastrointestinal cancer

**First Author, Year, Study Place**	**Data Collection Period**	**Study Design**	**Sample Size**	**Nutritional Assessment**	**Quality of Life Assessment**	**Groups being compared**	**Key results**	**Conclusion**
Tian J, 2009, China [20]	January 2007 to December 2007	Cross-sectional study	233 advanced stomach cancer	Daily calorie and protein intake using Food Frequency Survey Method and Food Composition Database, BMI, albumin	ECOG performance status	BMI <18 kg/m^2^ and > =18 kg/m^2^ Albumin <35 g/L and > =35 g/L Daily Calorie intake <2400 kcal and > =2400 kcal Daily Protein intake <70 g and > =70 g	The relative risk (95% confidence interval) was 1.16 (1.02–1.32) for low level of daily calorie intake versus normal level of daily calorie intake.	Low level of daily calorie intake may be the risk factor of poor performance status of the patients with advanced stomach cancer
Tian J, 2008, China [21]	January 2006 and June 2006	Cross-sectional study	113 esophagus, stomach, and colorectal	Daily calorie and protein intake using Food Frequency Survey Method and Food Composition Database, BMI, albumin	EORTC QLQ-C30	Calorie intake, BMI and albumin used as continuous variables	After age, sex, and stage of the disease were adjusted, patients with high daily intakes of calories and protein, as well as high level of albumin, had a significantly better quality of life.	Nutrition status 1 year after being discharged from hospitals may be associated with better QoL in patients with esophagus, stomach, and colon cancers
Correia M, 2007, Portugal [22]	December 2003 to November 2004	Prospective consecutive case series	48 with a recent (< 4 weeks) diagnosis of gastric cancer	1. Percentage of weight loss* 2. PG-SGA 3. BIA for FFMI 4. Hand Grip Dynamometry	EORTC-QLQ C30	**1. Weight Loss:** > 10% in the previous six months or > 5% in the last month & < 10% in the previous six months or < 5% in the last month. **2. PG-SGA** Well-nourished, mild malnutrition (MN) & severe MN. **3. Hand Grip Dynamometry:** Below 85% & above 85%	Malnutrition identified through PG-SGA, percentage of weight loss at 1 month, FFMI or dynamometry was positively associated to a worse QoL with the worst performance in all dimensions of QoL being attributed to those patients identified as malnourished by PG-SGA.	PG-SGA was correlated with the several dimensions for QoL evaluation.
Martin L, 2007, Sweden [23]	2 April 2001 to 30 October 2004	Prospective population-based cohort study	233 with esophageal or cardia cancer Adenocarcinoma cardia: n = 102; esophageal adenocarcinoma: n = 82; Oesophageal squamous cell carcinoma: n = 49	Postoperative weight change, measured as the difference in BMI between the time of tumor resection and 6 months later	1. EORTC QLQ-C30 2. QLQ-OES18	**Postoperative weight change –** **Six groups:** **Group I:** Stable or increased, **Group II:** decrease of 1–4%, **Group III:** 5–9% decrease, **Group IV:** 10–14% decrease, **Group V:** 15–19% decrease, **Group VI:** ≥ 20% decrease	Patients with a BMI decrease of at least 20 per cent experienced more appetite loss (mean score difference 26; P = 0·002), eating difficulties (mean score difference 18; P < 0·002) and odynophagia (mean score difference 12; P = 0·044) than patients without postoperative weight loss, whereas scores for dysphagia and gastro-oesophageal reflux were similar between these groups.	Malnutrition is a considerable problem after oesophagectomy, and is linked to appetite loss, eating difficulties and odynophagia.
Gupta D, 2006, USA [24]	March 2001 to June 2003	Retrospective	58 histologically confirmed stages III and IV colorectal cancer	1. Serum albumin, 2. Prealbumin, 3. serum Transferrin, 4. Phase angle by BIA 5. SGA	EORTC-QLQ C30	**Well nourished:** SGA-A (n = 34) & **Malnourished:** (SGA-B&C) (n = 24) All others were used as continuous variables.	**SGA:** Well-nourished patients had significantly better QoL scores in the global, physical, role function scales and fatigue, pain, insomnia, appetite loss, and constipation symptom scales. **Serum albumin, serum transferrin, and phase angle:** were significantly correlated with the physical and role function scales and fatigue and appetite loss symptom scales.	Malnutrition is associated with poor QoL, as measured by the QLQ-C30 in colorectal cancer.
Tian J, 2005, China [25]	April 2004 to May 2004	Retrospective	285 surgical stomach cancer	Daily calorie intake using Food Frequency Survey Method and Food Composition Database	3 QoL groups: bad (total score under 60), modest (total score within 60–80) and good (total score over 80)	Good, modest and bad quality of life	For both males and females, the daily nutrition intake among three groups, except vitamin C, were statistically different, which suggested that the patients who had a better nutritional status had a higher quality of life.	The nutritional status of the operated patients with stomach cancer may impact their QoL. Exercise for rehabilitation can whet the appetite of the patients and recover their body function, which in turn may improve QoL.
Andreyev HJN, 1998, UK [26]	April 1990 to March 1996	Retrospective	1555 tumors of oesophagus, stomach, pancreas, colon or rectum Oesophageal: n = 179; Gastric: n = 433; Pancreatic: n = 162; Colorectal: n = 781;	Weight loss at presentation	EORTC-QLQ-C30	With weight loss & no weight loss	Patients with weight loss at presentation had a mean quality of life score which was less than patients with no weight loss, especially in patients with gastric (P < 0.008), pancreatic and colorectal cancers (P < 0.0001) and also when all sites were combined. (P < 0.0001).	Patients with weight loss had a worse quality of life score.
O’Gorman P, 1998, UK [27]	NA	Prospective	119 gastrointestinal cancer Colorectal: n = 43; Esophageal: n = 27; Gastric: n = 38; Pancreatic: n = 11	Weight loss* * defined as loss of more than 5% pre-illness weight in the previous 6 months	1. EuroQol EQ-5D 2. EORTC QLQ-C30	Weight-stable (< 5% weight loss) (n = 22) & Weight-losing (> 5% weight loss) (n = 97)	**1. EuroQol EQ-5D –** Median (range) = 0.85 (0.03-1.00) & 0.52 (−0.26-1.00) respectively for weight-stable and weight-losing groups, p < 0.001. **2. EORTC QLQ-C30 –** The results in most subscales of the EORTC QLQ-C30 questionnaire were poorer in the weight-losing group (p < 0.01).	Weight loss and reduction of appetite are important related factors in lowering the quality of life of gastrointestinal cancer patients.

**Table 3:** Nutritional status and quality of life in lung cancer

**First Author, Year, Study Place**	**Data Collection Period**	**Study Design**	**Sample Size**	**Nutritional Assessment**	**Quality of Life Assessment**	**Groups being compared**	**Key results**	**Conclusion**
Scott HR, 2003, UK [28]	NA	Prospective	106 inoperable NSCLC (stage III and IV) **By stage –** Stage III: n = 78 Stage IV: n = 28	Weight loss* * defined as loss of more than 5% pre-illness weight in the previous 6 months	EORTC-QLQ-C30	**Weight-stable** (< 5% weight loss) (n = 61) &**Weight-losing** (> 5% weight loss) (n = 45)	**(a) Global QoL:** Median (range) = 50 (0–100) & 33.3 (0–66.7) respectively for weight-stable & weight-losing groups, p = 0.027. **(b) Symptom scores:** Fatigue (P < 0.05) and pain (P < 0.01) were significantly greater in the weight-losing group. i. Fatigue: – Median (range) = 55.6 (0–100) & 66.7 (0–100) respectively for weight-stable & weight-losing groups, p = 0.044. ii. Pain: – Median (range) = 16.7 (0–100) & 41.7 (0–100) respectively for weight-stable & weight-losing groups, p = 0.007.	Weight loss has an impact on different aspects of quality of life.

**Table 4:** Nutritional status and quality of life in gynecological cancer

**First Author, Year, Study Place**	**Data Collection Period**	**Study Design**	**Sample Size**	**Nutritional Assessment**	**Quality of Life Assessment**	**Groups being compared**	**Key results**	**Conclusion**
Gil KM, 2007, USA [29]	January 2001 to July 2004	Prospective longitudinal study, consecutive case series	157 requiring surgery for a pelvic mass or a positive endometrial biopsy (endometrial cancer) Ovarian cancer: n = 33 Endometrial cancer: n = 45 Benign adnexal mass: n = 79	BMI (kg/m^2^)	1. SF-36 for General Health Status 2. FACT-G	BMI was used as a continuous variable	Univariate: Increasing BMI was negatively correlated with physical, social and functional well being. Multivariate: BMI continued to be a significant independent variable included in the model for social well-being, p = 0.03.	BMI was significantly associated with QoL. As treatment options become more complex, these variables are likely to be of increasing importance in evaluating treatment effects on QoL.

**Table 5:** Nutritional status and quality of life in heterogeneous cancer population

**First Author, Year, Study Place**	**Data Collection Period**	**Study Design**	**Sample Size**	**Nutritional Assessment**	**Quality of Life Assessment**	**Groups being compared**	**Key results**	**Conclusion**
Norman K, 2010, Germany [30]	NA	Prospective cross-sectional	189 Gastrointestinal: 103; Head and Neck: 30; Urinary Tract: 8; Gynecologic: 21; Others: 13	SGA	EORTC-QLQ-C30	SGA-A: Well nourished (n = 109) SGA-B: Moderately malnourished &SGA-C: Severely malnourished (n = 80)	Most QoL functional scales were significantly reduced in malnutrition and the majority of symptom scales were higher in the malnourished patients. Malnutrition emerged as an independent determinant for functional status (estimated effect size 19.4%, p < 0.001) next to age and gender, which were the strongest predictors.	Malnutrition is a disease independent risk factor for reduced muscle strength and functional status in cancer patients.
Norman K, 2010, Germany [10]	December 2006 to June 2007	Prospective consecutive case series	399 with solid or hematologic tumor disease Gastrointestinal: n = 149; Head and neck or lung: n = 71; Urogenital: n = 23; Gynecologic: n = 35; Neuroendocrine, adrenal, thyroid: n = 30; Others: n = 20; Hematologic disease: n = 71	1. SGA 2. Phase angle determined by BIA	EORTC-QLQ-C30	**1. SGA –** SGA-A: Well nourished (n = 167) SGA-B: Moderately malnourished (n = 132) &SGA-C: Severely malnourished (n = 100). **2. BIA –** Below fifth percentile (n = 191) & above fifth percentile (n = 208) of the phase angle	Univariate: All function scales of the EORTC quality-of-life questionnaire apart from emotional function were significantly impaired in patients with a phase angle below the fifth reference percentile, and among the symptom scale, fatigue, nausea and vomiting, pain, dyspnea, appetite loss, and constipation were increased. Multivariate: The standardized phase angle was an independent predictor of muscle function as were sex, age, and SGA in a GLM regression model and an independent predictor for EORTC global function score next to SGA, BMI, handgrip strength, and age.	The standardized phase angle is an independent predictor for impaired functional and nutritional status than are malnutrition and disease severity in cancer.
Shahmoradi N, 2009, Malaysia [5]	November 2008 to April 2009	Prospective	61 Colon: n = 8; Rectum: n = 8; Breast: n = 11; Lung: n = 7; Stomach: n = 4; Prostate: n = 3; Kidney: n = 3; Nasopharynx: n = 3; Leukemia: n = 3; Liver: n = 2 Brain: n = 2; Cevix uteri: n = 1; Ovary: n = 1; Pancreas: n = 1; Other: n = 4	PG-SGA	HQLI	Well-nourished, Severely malnourished & Moderately malnourished	Univariate: The PG-SGA score was significantly correlated to total quality of life score (p = 0.000). PG-SGA score alone was able to explain 38% of the total variation in total quality of life score. Multivariate: PG-SGA score showed significant correlation with psychophysiological well-being (p = 0.000), functional well-being (p = 0.000) and social/spiritual well-being (p = 0.040). PG-SGA score is able to explain 36.9%, 41.8% and 7% of the total variation in psychophysiological, functional and social/spiritual wellbeing, respectively.	Advanced cancer patients with poor nutritional status have a diminished quality of life. There is a need for a comprehensive nutritional intervention for improving nutritional status and quality of life in terminally ill cancer patients under hospice care.
Tong H, 2009, Australia [3]	Data collection concluded in 2000, primary data analysis by 2001	Prospective observational longitudinal study	219 solid and hematological Head neck: n = 7;Gastrointestinal: n = 47; Breast: n = 63; lung: n = 15; urinary: n = 31; Soft tissue–skin–brain–CNS: n = 7; Primary unknown: n = 3; Hematological: n = 46	PG-SGA	Global QoL was measured using Life Satisfaction Scale	Both PG-SGA & QoL are used as continuous variables	A small to medium negative correlation was found between PG-SGA scores and life satisfaction scores across all time points. **1. At baseline (n = 218):** r = −0.224, p = 0.001 **2. At 6 months (n = 196):** r = −0.350, p < 0.001 **3. At 12 months (n = 157):** r = −0.288, p < 0.001).	Nutrition impact symptoms were commonly experienced, even 12 months following commencement of chemotherapy, and were associated with poorer QoL and performance status.
Nourissat A, 2008, France [31]	Over 2 weeks	Transversal observational study	883 evolving cancer s **Males (n = 434)** Lung: n = 105; Colorectal: n = 84; Prostate: n = 67 **Females (n = 449)** Breast: n = 194; Colorectal: n = 79;Ovary: n = 33	Weight loss	EORTC-QLQ C30	**Weight loss < 10%** (n = 622) &**Weight loss ≥ 10%** (n = 261)	(a) Mean Global QoL score = 62.8 & 48.8 respectively for weight loss < 10% & ≥ 10%, p < 0.001. (b) Physical, functional, emotional, cognitive and social functions were significantly higher in weight loss < 10% group. Symptom scores were also lower for fatigue, nausea, vomiting, pain, dyspnea, loss of appetite, constipation and diarrhea.	To improve QoL in patients with cancer, a nutritional intervention should be implemented as soon as cancer is diagnosed. The nutritional therapy should form part of the integral oncological support.
Trabal J, 2006, Spain [6]	April 2004 to September 2004	Descriptive cross-sectional study, consecutive case series	50 non-terminal cancer Lung: n = 20; Breast: n = 7; Gynecologic: n = 6; Esophagus: n = 4; Others: n = 13	1. BMI 2. Percentage of usual weight 3. Ideal weight percentage, 4. Percentage weight loss 5. TSF 6. Mid-upper arm circumference 7. Serum albumin 8. Prealbumin 9. Total proteins	EORTC QLQ-C30	Protein intake < 0.9 g/kg/d & ≥ 0.9 g/kg/d	1. Patients with hypo albuminemia reported more problems with diarrhea (p = 0.05). 2. Protein intake below 0.9 g/kg was associated to a poorer perception on physical functioning (p = 0.01), and fatigue was close to significance (p = 0.058). 3. No significant differences were found regarding caloric intake though, being fatigue (p = 0.06) the closest relation. 4. No other nutritional parameters, like percentage of weight loss, were statistically related to changes in QoL.	Nutrition is only one of the factors that influence QoL in cancer patients, but nutritional evaluation of cancer patients needs to be improved and individualized nutritional counseling should be done, so as to offer better treatment of symptoms and to improve patients’ QoL.
Ravasco P, 2004, Portugal [32]	July 2000 to September 2002	Prospective, cross-sectional, consecutive case series	271 Head and neck Base of tongue: n = 11; Salivary gland: n = 6; Tonsil: n = 4; Nasopharynx: n = 11; Oropharynx: n = 22; Larynx: n = 33; Oesophagus: n = 14; Stomach: n = 26; Colorectum: n = 144	Percentage weight loss over the previous 6 months	EORTC-QLQ C30	≥ 10% weight loss & < 10% weight loss over the previous 6 months	Malnutrition was associated with poorer function scales and with some symptoms: global QoL (P = 0.05), physical (P = 0.01), role (P = 0.02), cognitive (P = 0.02), emotional (P = 0.01) and social (P = 0.01); anorexia (P = 0.001), increased fatigue (P = 0.03), dyspnea, insomnia and diarrhea (P = 0.04).	Although cancer stage was the major determinant of patients’ QoL globally, there were some diagnoses for which the impact of nutritional deterioration combined with deficiencies in nutritional intake may be more important than the stage of the disease process.
Isenring E, 2003, Australia [2]	Over a 1 year period	Prospective longitudinal	60 ambulatory patients receiving radiation therapy to the head, neck, rectal or abdominal area	PG-SGA	EORTC-QLQ C30	well-nourished (n = 39) malnourished (n = 21) PG-SGA scores and QoL used as continuous variables	**(A) At baseline –** Correlation between PG-SGA score and global QoL r = − 0.66, P < 0.001 **(B) After 4 weeks of radiotherapy –** Correlation between PG-SGA score and global QoL r = − 0.61, P < 0.001 **(C) Correlation between the change in PG-SGA score and change in global QoL** r = − 0.55, P < 0.001 26% of the variation of change in QoL was explained by change in PG-SGA score (P = 0.001). A change in PGSGA score of 9 resulted in a change of 17 in the QoL score.	The scored PG-SGA is a nutrition assessment tool that identifies malnutrition in ambulatory oncology patients receiving radiotherapy and can be used to predict the magnitude of change in QoL.
Ravasco P, 2003, Portugal [7]	July 2000 to February 2001	Prospective longitudinal study, Consecutive case series	125 **HR: High Risk** Oesophagus: n = 6;Stomach: n = 5; Colorectal: n = 46; Base of the tongue: n = 3; Salivary gland: n = 1; Tonsil: n = 2 Nasopharynx: n = 3; Oropharynx: n = 3; Larynx: n = 11 **LR: Low Risk** Prostate: n = 21; Breast: n = 7; Lung : n = 5; Brain: n = 4; Gallbladder: n = 6; Uterus: n = 2	SGA	1. EUROQOL 2. EORTC-QLQ-C30 QoL used as a continuous variable	Normal, moderate and severe malnutrition groups	**(A) EUROQOL –** 1. At baseline: In HR patients, baseline malnutrition was associated with lower self reported health status (SRHS) (P = 0.002). 2. At the end of Radiotherapy: Improved nutritional status was associated with higher SRHS (P = 0.03). **(B) EORTC-QLQ-C30 –** (a) At baseline: In HR patients, malnutrition associated with worse function scales: global QoL (P = 0.05), physical (P = 0.01), role (P = 0.02), cognitive (P = 0.02), emotional (P = 0.01) and social (P = 0.01) as well as symptoms: poor appetite (P = 0.001) or increased fatigue (P = 0.03) (b) At the end of Radiotherapy: All associations with function scales were also present at the end of treatment: global QoL (P = 0.01), physical (P = 0.02), role (P = 0.02), cognitive (P = 0.03), emotional (P = 0.01) and social (P = 0.04). In LR patients, nutritional parameters were not significantly associated with QoL dimensions.	Malnutrition as assessed by SGA was associated with a worse QoL in high risk patients.
Ovesen L, 1993, Denmark [33]	In 1989	Prospective	104 biopsy-proven breast cancer, ovarian cancer, or small cell lung cancer. Breast: n = 19; Ovarian: n = 47; Small cell lung: n = 38	Unintentional weight loss* *defined as weight loss within recent 3 months	1. GHQ 2. QL	**Weight-losing group (− weight loss):** weight loss of ≥ 5% of habitual body weight (n = 56) & **Weight-stable group (+ weight loss):** weight loss of < 5% of habitual body weight (n = 48)	General health, as assessed by the GHQ score, was rated significantly worse by patients with weight loss than by weight-stable patients. Similarly, the scores on the social functioning and the outlook/happiness subscales indicated significantly lower quality of life for the patients with weight loss, and this result was confirmed by a significant group difference on the modified QL.	Many ambulatory cancer patients do not eat enough to maintain weight and that even a moderate weight loss is associated with psychological distress and lower quality of life.

### Data items

Instead of providing aggregate quality scores, we assessed the quality of individual studies by reporting the key components of study designs. The following information was extracted for each article: first author, year of publication, study place, data collection period, study design, sample size, nutritional assessment method, QoL assessment method, nutritional groups being compared, key results and conclusions.

### Synthesis of results

The authors summarized all studies reviewed in this paper in separate tables based on the cancer type. Within each table, studies are arranged chronologically by the year of publication, beginning with the most recently published study.

## Results

### Study selection

**Figure 1:**
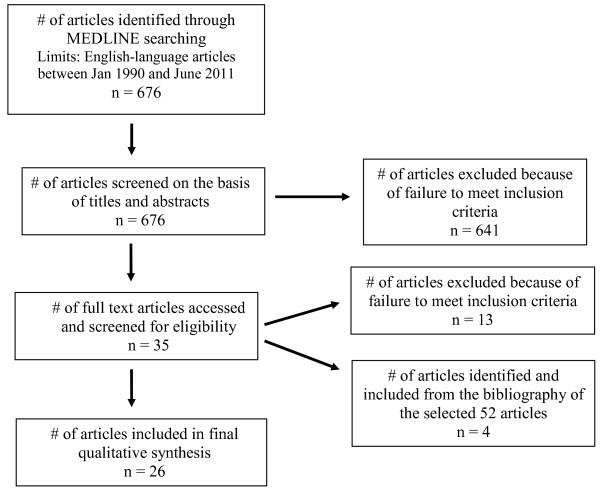
Flow-chart depicting MEDLINE search results.

The MEDLINE search identified a total of 676 articles based on different combinations of search terms described above. The authors reviewed the titles and abstracts of these 676 articles to identify the relevant articles based on the selection criteria described above. Of the 676 original articles, the authors excluded 641 from this review because it was clear from the abstracts that these papers did not meet the selection criteria. The authors then obtained the full texts of the remaining 35 articles for review. Thirteen of the 35 articles failed to meet the selection criteria and were excluded. The authors then identified 4 additional articles from the bibliographies of the selected 35 articles. Twenty-six articles were then included in the final review for this manuscript. Figure 1 is a flow chart that describes this process and its results.

### Characteristics of included studies

All 26 articles included in this review were published in English. The authors extracted the following data from each included study: first author, year of publication, study place, data collection period, study design, sample size, nutritional assessment method, QoL assessment method, nutritional groups being compared, key results and conclusions.

### Synthesis of results

#### Nutritional status and QoL in head and neck cancer

Table 1 describes studies exploring the relationship between nutritional status and QoL in patients with head and neck cancer. Of the six studies, one was a retrospective consecutive case series[17], one was cross-sectional[16] and four were prospective.[8,12,18,19] The sample size in these studies ranged from 36[17] to a maximum of 115[16] patients. The study populations included locally advanced head and neck cancers, cancers of the oropharynx, oral cavity/nasopharynx, larynx, maxillary sinuses/submandibular glands/pharyngeal and hyopharyngeal regions. Weight loss was the most commonly used nutritional assessment method, which was measured exclusively[18,19] or in combination with PG-SGA[8] or together with anthropometric measurements [AMC and TSF], weight index (WI), body mass index (BMI) and serum albumin (S-alb) level.[12] One of the studies assessed nutritional status in terms of the drop in BMI over a period of 12 months.[17] The studies also employed various tools for assessment of QoL, which included the EORTC-QLQ-C30, either singly [8] or together with a head and neck specific module EORTC-QLQ-H&N35 – either alone[18] or in combination with Hospital Anxiety and Depression Scale (HADS).[19] One of the studies used the EORTC-QLQ-C30 together with a head and neck module developed in Norway[12] while another used the University of Washington Head and Neck Disease-Specific Measure (UW-QOL).[17] Weight loss was the principal criterion used to categorize the study population into comparison groups. Two studies categorized the patients based on their weight loss percentage (≥ 10% and < 10%)[18,19] while another study categorized its sample population based on unintentional weight loss of < 5% (non-malnourished) and ≥ 5% (malnourished) respectively.[8] Still another study used the change in BMI as a continuous variable[17], and another grouped its study population based on malnourished vs. normal nutrition, weight loss vs. no weight loss and negative vs. positive energy balance.[12] All six studies concluded that better nutritional status was positively associated with better QoL in the study patients. Interestingly, 3 studies[12,17,18] found an association between weight loss & swallowing function. Swallowing difficulty can cause weight loss, and as people lose weight, swallowing function can be further compromised due to muscle function loss and contribute to decline in QoL.

#### Nutritional status and QoL in gastrointestinal cancer

Table 2 describes studies that explored the association between nutritional status and QoL in patients with gastrointestinal cancer. Of the eight studies included in this table, three were retrospective[24-26], two were cross-sectional[20,21] and three were prospective[22,23,27]. The sample size ranged from 48[22] to 1,555[26] patients. The population under study had been diagnosed with cancer of the gastric/gastrointestinal region, esophagus or cardia, colorectal region or pancreas. The most commonly used nutritional assessment methods were the percentage of weight loss, either alone[26,27,34] or in combination with PG-SGA, phase angle measured in terms of BIA or Fat Free Mass Index (FFMI) and hand-grip dynamometry.[22] One study assessed nutritional status in terms of postoperative weight loss measured by the difference in BMI.[23] Another study used several different assessment measures including serum albumin, prealbumin, serum transferrin, phase angle by BIA and SGA.[24] The studies also employed various tools to assess the patients’ QoL. The most commonly used was the EORTC-QLQ-C30, either exclusively[22,24,26,34] or together with other tools like EuroQol EQ-5D[27] or together with an esophageal cancer specific module EORTC OES-18.[23] Weight loss was the primary criterion for segregation of the study population into groups for comparison. The studies used a variety of criteria to divide the patients into groups, including well-nourished vs. malnourished individuals[24], weight-stable vs. weight-losing patients[27], and patients with and without weight loss.[26] One study compared patient groups based on a BMI decline of ≥ 20% and < 20%.[23] Another study segregated its population based on several parameters that included > 10% weight loss in the previous six months or > 5% weight loss in the last month and < 10% weight loss in the previous six months or < 5% weight loss in the last month, or as well-nourished, moderately malnourished and severely malnourished (in terms of PG-SGA scores), or grip strength below 85% & above 85% (based on gand-grip dynamometry).[22] All 8 studies concluded that better nutritional status was positively associated with better QoL in the study patients.

#### Nutritional status and QoL in lung cancer

Table 3 describes the lone prospective study that assessed the association between nutritional status and QoL in 106 stage III and IV patients who had been diagnosed with inoperable non-small cell lung cancer (NSCLC). The patients’ nutritional status was assessed in terms of weight loss while QoL was assessed using EORTC-QLQ-C30. The sample population was subdivided into two groups: weight-stable and weight-losing based on whether they had lost < 5% or > 5% weight. The weight-stable patients reported better global QoL and less fatigue and pain than the weight-losing group.[28]

#### Nutritional status and QoL in gynecological cancer

Table 4 describes the lone prospective study that assessed the association between nutritional status and QoL in 157 gynecological cancer patients who required surgery for a pelvic mass or who had a positive endometrial biopsy. Nutritional status was assessed in terms of BMI (used as a continuous variable) while QoL was assessed using the SF-36 and FACT-G questionnaires. More than 70% of the patients were either overweight or obese. The study showed that higher BMI was negatively correlated with QoL upon both univariate and multivariate analyses.[29]

#### Nutritional status and QoL in heterogeneous cancer patient populations

Table 5 describes the relationship between nutritional status and QoL in heterogeneous cancer patient populations. Of a total of 10 studies listed in this table, one was a transverse observational longitudinal study[31], three were prospective longitudinal[2,3,7], one was descriptive cross-sectional[6] and two were prospective cross-sectional.[30,32] The remaining three studies were prospective.[5,10,33] The sample size ranged from 50[6] to 883 patients.[31] The population studied included patients with solid or hematologic tumor disease; evolving cancer at different management stages; non-terminal cancer patients; ambulatory patients with head and neck, esophageal, stomach and colorectal cancer; ambulatory patients who were receiving radiation therapy to the head, neck, rectal or abdominal area; patients with tumors of the head and neck, gastrointestinal tract (high-risk: HR), prostate, breast, lung, brain, gallbladder or uterus (low-risk: LR); or biopsy-proven breast, ovarian, or small cell lung cancer. The patients’ nutritional status was most commonly assessed with the SGA, either singly [7] or in combination with phase angle determined by BIA[10] or in the form of PG-SGA scores.[2,3,5] Three studies used weight loss as the nutritional assessment tool[31-33] while another study assessed nutritional status using multiple parameters, including BMI, percentage of usual weight, ideal weight percentage of ideal weight, percentage of weight loss, triceps skinfold thickness, mid-upper arm circumference, serum albumin, prealbumin, total proteins and total cholesterol.[6] The principal tool used for the measurement of QoL was EORTC-QLQ-C30, either alone[2,5,6,31,32] or in combination with EuroQoL.[7] The remaining three studies used different QoL tools, including the Life Satisfaction Scale[3], Hospice Quality of Life Index (HQLI)[5], and the General Health Questionnaire (GHQ) together with the Quality of Life Index (QL).[33] Two studies classified their sample population into three distinct categories: well nourished, moderately malnourished and severely malnourished on the basis of SGA/PG-SGA scores.[5,7] One study used PG-SGA scores as a continuous variable [3] while another study treated PG-SGA scores as a continuous variable despite having categorized its patients as being well nourished and malnourished.[2] Three studies divided their sample into two categories each based on percentage of weight loss using the cut-off criteria of 10% (i.e. < 10% & > 10% weight loss)[10,32] and 5% [i.e. ≥ 5% (weight-losing or –weight loss) and < 5% (weight-stable or + weight loss)][33] respectively. Another study grouped its sample population based on the quantity of protein intake (< 0.9 g/kg/d & ≥ 0.9 g/kg/d).[6] One study used two criteria to categorize its sample population: SGA levels and BIA. The three groups included well nourished, moderately malnourished and severely malnourished based on SGA. Patients were also categorized in two groups: below and above the fifth percentile of the phase angle as measured by BIA.[10] Of the 10 studies, one showed that nutritional status was significantly associated with QoL only for high-risk patients. This was true when both EuroQoL and EORTC-QLQ-C30 were used as tools for measurement of QoL.[7] Another study concluded that nutritional status was not significantly associated with QoL.[6] The remaining 8 studies showed a positive correlation between nutritional status and QoL[2,3,5,10,31-34].

## Conclusion

Patient QoL is an extremely important outcome measure for cancer patients. How patients feel, physically and emotionally, while they are fighting cancer can have an enormous effect on their ability to carry out normal daily functions as well as on their interpersonal relationships and their ability to work.

Cancer and its treatment affects the nutritional status of patients by altering their metabolic function and reducing their food intake.[5,6] Research has proven that malnutrition is a predictor of morbidity in advanced cancer; therefore, malnutrition is also likely to assume a significant role in patients’ QoL.[24] The present study aimed to systematically review the relationship between nutritional status and QoL in cancer patients. A total of 26 original studies were included in this review. Of the 26 studies, 6 investigated the correlation in head and neck cancer patients, 8 in gastrointestinal cancer patients, 1 in lung cancer patients, 1 in gynecologic cancer patients and 10 in heterogeneous cancer populations.

Better nutritional status was associated with better QoL in all 6 studies of head and neck cancer patients with each study identifying different reasons for the correlation. One study reported that weight loss in some patients was related to loss of speech and swallowing capabilities, which may have affected patients’ ability to take food by mouth.[17] Another study concluded that weight loss of more than 10% had significant impact on QoL scores at time of diagnosis and that it seemed to significantly worsen global QoL, fatigue and pain. The same study advised that patient weight loss should be limited as much as possible starting at diagnosis and continuing until six months after treatment.[18] Another study showed that patients in the ≥10% weight-loss group reported extreme problems (>80 points) with dry mouth and sticky saliva at 3 months, 1 year and 3 years after diagnosis. This effect was attributed, on the basis of previous studies, to the fact that this patient population was comprised of more people who lived alone, more smokers/ex-smokers, a higher percentage of patients with stage III to IV disease (95% vs 50%), a higher percentage of patients with pharyngeal cancer, and more patients who received chemotherapy.[19] In summary, these 8 studies indicate that poor nutritional status, measured primarily using weight loss, was a strong predictor of patient QoL, measured primarily through EORTC QLQ-C30, in head and neck cancer patients.

All 8 studies that explored the association between nutritional status and QoL in gastrointestinal cancer patients concluded that better nutritional status was associated with better QoL. One study theorized that an inflammatory response may contribute to weight loss in advanced gastrointestinal cancer patients by increasing energy expenditure and the turnover of specific amino acids, which reprioritizes the body’s protein metabolism away from peripheral tissues and toward the liver. This process appears to contribute to the preferential loss of protein (in particular, skeletal muscle) in such patients. This in turn may be a cause of appetite loss and lowered QoL in gastrointestinal cancer patients.[27]

The lone study that reported a significant relationship between nutritional status and QoL in lung cancer patients speculated that the relationship between pain and more than 5% prior weight loss may simply be a result of more advanced disease, as there were a greater number of weight loss patients in this group who had been diagnosed with stage IV disease.[28] The lone study that explored the association between nutritional status and QoL in gynecological cancer patients indicated that nutritional status (in terms of BMI) was significantly associated with QoL.[29] More than 70% of the patients were either overweight or obese suggesting that obesity is another form of malnutrition that is often overlooked in clinical settings, and can have a negative impact on patient QoL.

Of the 10 studies that investigated the relationship between nutritional status and QoL in a heterogeneous cancer population, eight concluded that nutritional status was significantly associated with QoL, one found nutritional status to be significantly associated with QoL only for high-risk patients[7], and one found no association between the two.[6] Of the eight studies reporting significant association, one reported that although an association between malnutrition and impaired QoL was observed in all sub-groups of patients, it was not possible to identify which was the cause and which was the consequence: weight loss or QoL. The authors attributed this issue to the study design (transversal study).[31] Another study also inferred that it was not possible to conclude which came first – insufficient food intake, decreased QoL or weight loss – although the authors did establish that the three variables were interdependent.[33].

Overall, among the 26 studies reviewed in this article, 24 concluded that better nutritional status was associated with better QoL, one study showed that better nutritional status was associated with better QoL only in high-risk patients, and one concluded that there was no association between nutritional status and QoL.

The majority of the studies reviewed in this article used weight loss (expressed as weight loss or unintentional weight loss or percentage of weight loss) as the tool for assessment of nutritional status, either exclusively[18,19,26-28,31-33] or in combination with other methods.[8,12,22] The results described by these papers suggest that weight loss is a good prognosticator of QoL irrespective of the type of cancer for a number of reasons. One, weight loss is a common feature of advanced cancer due to patient distress and loss of independence.[27] Weight loss is a known cause of morbidity and mortality in cancer patients that also decreases patient tolerance to both radiotherapy and chemotherapy.[19] Weight loss as low as 5% can alter important, measurable physiological parameters such as immune response, lung and cardiac function tests and autonomic autoregulation.[26] More than 10% weight loss at diagnosis has a great impact on QoL scores.[18] A total weight loss of ≥ 20% significantly correlates with treatment interruption, infections, early mortality, hospital re-admission rate after treatment completion as well as survival.[8] Recent work suggests that an inflammatory response might contribute to the weight loss in advanced gastrointestinal cancer patients by increasing energy expenditure and the turnover of specific amino acids, which reprioritizes the body’s protein metabolism away from peripheral tissues and toward the liver. This process apparently contributes to the preferential loss of protein (in particular, skeletal muscle) in such patients. The reprioritization of metabolism may also impact patient appetite and, along with it, the QoL of gastrointestinal cancer patients.[27] There is evidence in the literature reviewed that the use of “percentage weight loss since the start of the illness” is a relatively objective measure, although the patient’s usual or “normal” weight is often only approximately known. On the other hand, percentage weight loss does not appear to account for the kinetics of weight loss, presence of edemas, water retention and clinical-biological effects.[31] On the contrary, SGA is the only malnutrition screening tool recommended by the ASPEN board of directors.[35] SGA is a simple, easy-to-apply and cost-effective method that has been validated for diverse groups of patients. SGA is one of the better available assessment methods, not only because it is patient centred and incorporates clinical history and physical examination, but also does not require laboratory testing or medical imaging exams.[36,37] Reliable SGA grading, however, depends on collection of correct history and physical observation and requires a skilled dietician to carry out the assessment. Nutrition assessment tools such as the scored PG-SGA enable nutritional status to be assessed quickly, nutrition impact symptoms identified and appropriate nutrition support implemented. An advantage of the PG-SGA as a nutrition assessment tool is that the score can be used as an outcome measure in nutrition intervention studies as it may be more sensitive to changes in nutritional status than the global SGA rating.[38] Also, by performing serial measurements, the change in the PG-SGA score may be used to demonstrate subtle changes in nutritional status.

The majority of the studies reviewed here used EORTC-QLQ-C30 to assess patient QoL, either exclusively[2,8,10,22,24,26,28,31,32] or in combination with other QoL tools.[7,12,18,19,23,27] The EORTC QLQ-C30 questionnaire is a validated instrument for assessing QoL in patients with cancer.[31] It is usually completed by self-assessment[12,27] and covers more items and scales, identifies more domains and specific complaints, and assesses cancer and radiotherapy specific symptoms, and is, therefore, a more comprehensive and sensible measure than some others.

Collectively, the studies reviewed in this article suffered from certain limitations. Three studies involved small sample sizes, which made comparisons and statistical analyses difficult.[5,17,24] Non-responders contributed toward bias in one study[17], while another made no assessment of inter-rater reliability of the users of SGA and BIA. These studies minimized this bias by using only BIA-trained dieticians.[24] Two studies reported exclusion of patients with physical, cognitive, language or emotional problems that prevented them from completing the respective QoL questionnaires.[2,5] Another study was a secondary analysis and was not designed as a nutrition trial. As a result, some of the nutritional parameters included in the survey were limited. Also, there was significant attrition between T2 and T3 and, apparently, more stage IV patients were lost to attrition. Thus, the prevalence of nutrition impact symptoms in these patients may have been underreported.[3] One study reported that its outcome data may have been misclassified, but then ruled out the probability of error on the grounds that 1) the analysis of self-reported preoperative body weight compared with body weight measured by surgical staff before operation showed good validity and 2) that the questionnaires covering nutritional issues had been previously validated.[23]

Like most other systematic reviews of the literature, this review suffers from potential publication bias. In general, this bias exists because studies that report positive associations are more likely to be published. Therefore, it is possible that studies containing valuable data may not have been published and have gone undetected. Since we restricted this systematic review to studies published in English, it is possible that language bias may have affected our conclusions. Finally, our review simply focused on the relationship between nutritional status and QoL in cancer, which does not by any means imply causation. As a result, a logical next step would be to systematically review the available literature, if any, to investigate whether nutritional intervention can have a favorable impact on QoL outcomes in cancer patients. Despite these limitations, our review and analysis of the extensive available literature demonstrates a strong association between nutritional status and QoL in cancer.

Also as a result of our review, we have identified new avenues for further research in this area. One is to identify the best management practices for timing of nutritional assessment and intervention in cancer patients as, currently, there is no consensus on how to manage patients based on any of the nutritional metrics reviewed here. Nonetheless, this review of the literature provides a strong rationale for devising such standards of practice and testing their value in controlled clinical studies. All clinical manifestations of malnutrition should be included, as well as specific situations where a causative relationship with QoL is apparent.

Our review of the current literature supports the hypothesis that nutritional status is a strong predictor of QoL in cancer patients. It also supports an approach to cancer treatment that takes all aspects of the patient’s life into account. Further, the current literature supports the implementation of the ASPEN guidelines for oncology patients, which include nutritional screening, assessment, and intervention as appropriate. Correcting malnutrition in cancer patients can have a significant positive impact on their QoL.

## Competing interests

The authors declare that they have no competing interests.

## Authors’ contributions

CGL and DG participated in concept, design, research synthesis and writing. CAL, MM and PGV participated in concept, writing, critical input and revision. All authors read and approved the final manuscript.
